# Integrative structural insights into the IgG-FcRn interactions revealed by engineered FcRn-immobilized affinity chromatography

**DOI:** 10.1038/s42003-026-09789-3

**Published:** 2026-03-03

**Authors:** Masato Kiyoshi, Takuo Suzuki, Naruaki Inoue, Ryoko Otake, Tatsuya Yumoto, Linko Hirono, Yosuke Terao, Hiroko Shibata, Teruhiko Ide, Satoru Nagatoishi, Kouhei Tsumoto, Akiko Ishii-Watabe

**Affiliations:** 1 Division of Biological Chemistry and Biologicals, National Institute of Health Sciences, Kawasaki, Kanagawa Japan; 2 Life Science Research Laboratory, Tosoh Corporation, Ayase, Kanagawa Japan; 3 The Institute of Medical Science, The University of Tokyo, Tokyo, Minato-ku Japan; 4 The Department of Bioengineering, School of Engineering, The University of Tokyo, Tokyo, Bunkyo-ku Japan

**Keywords:** Structural biology, Antibody therapy

## Abstract

Molecular interactions between therapeutic antibodies and neonatal Fc receptor (FcRn) are major contributors to the long serum half-life of antibodies. Therefore, the in vitro affinity of IgG for FcRn is an important indicator of in vivo pharmacokinetic properties. However, the molecular basis of IgG-FcRn interactions is not fully understood from experimental and structural perspectives. Affinity evaluation using surface plasmon resonance is difficult. The molecular mechanism by which domains other than Fc or surface charges affect FcRn binding remains unclear. In this study, we developed an engineered FcRn-immobilized column for affinity chromatography. We analyzed various types of antibodies to link physicochemical characteristics and FcRn affinity. Furthermore, analysis using charge-engineered mutants indicated that the lateral surface of L chain also affects the FcRn affinity. Finally, we presented a structural model of the IgG-FcRn complex to discuss the molecular mechanism of how the charges at L chain affect the FcRn affinity.

## Abstract (web summary)

The authors developed an engineered FcRnimmobilized column. Analysis using the column revealed structural insights into IgG–FcRn interactions. Based on the results, a model of the IgG–FcRn complex on the membrane surface was proposed.

## Introduction

Therapeutic antibody drugs have proven to be one of the most important therapeutic modalities for the treatment of a wide range of diseases. Over the past three decades, the number of therapeutic antibodies in clinical use has increased dramatically^1^. Moreover, evolving biotechnologies have enabled scientists worldwide to enhance the therapeutic efficacy of these drugs. Next-generation, engineered therapeutic antibody drugs, such as antibody drug conjugates (ADCs), bispecific antibodies, and Fc-engineered antibodies, have delivered remarkable breakthroughs for the treatment of intractable diseases^2–4^.

IgG1 is the predominant molecular format of therapeutic antibody drugs. IgG1 comprises two Fab domains and an Fc domain. The Fc domain, in particular, has been intensively engineered. This is because the Fc domain governs the multifaceted characteristics of therapeutic antibodies, such as Fc-mediated effector functions (antibody-dependent cell-mediated cytotoxicity (ADCC), antibody-dependent cell-mediated phagocytosis (ADCP), complement-dependent cytotoxicity (CDC)), and a long serum half-life in patients.

The long serum half-life of therapeutic antibodies in patients, that is a pharmacokinetic (PK) property, has a great impact on therapeutic efficacy. Estimating PK properties of therapeutic antibody in humans remains challenging, since multiple parameters—such as FcRn affinity of the antibody, administration dose, administration route, patient body weight, properties of target molecules, and underlying conditions—affect PK properties. Furthermore, multiple mechanisms are involved in the regulation of the PK properties of administered therapeutic antibodies, including phagocytosis by the reticuloendothelial system (phagocytic immune cells such as macrophages and monocytes), target (antigen)-mediated internalization, and target-independent pinocytosis^5–7^. Importantly, the neonatal Fc receptor (FcRn) plays a key role in the pinocytosis-mediated antibody clearance pathway because of its function of protection and recycling of IgG^8,9^. Human FcRn is located on diverse cell types, including epithelial cells, endothelial cells, and immune cells. FcRn comprises an MHC-class-I-like molecule and a β_2_-microglobulin (β_2_m). FcRn plays pivotal roles in IgG homeostasis, albumin homeostasis, maternal IgG transport, and antigen presentation. Notably, the interaction between IgG and FcRn is pH-dependent^10^. At acidic pH, IgG binds to FcRn with moderate affinity, whereas at neutral pH, the affinity is absent. In endosomes, only FcRn-captured IgG is protected from lysosomal degradation at acidic pH and can be recycled back into the blood circulation at neutral pH. To predict PK properties of therapeutic antibodies in humans, in vivo studies using human FcRn transgenic mice has been widely used. However, such studies could pose a few issues, including non-specific cellular uptake and anti-drug antibody (ADA) in some cases^11–14^. Therefore, it is crucially important to first establish a thorough molecular-level understanding of in vitro FcRn affinity as a critical element in predicting in vivo PK properties.

Notably, the molecular basis of the IgG-FcRn interaction is not fully understood from experimental and structural perspectives. From an experimental perspective, the affinity evaluation of IgG-FcRn interaction is known to be difficult for the following reasons: (1) the binding stoichiometry between IgG and FcRn is not 1:1, thus the surface plasmon resonance (SPR) results are inconsistent depending on the FcRn-immobilization level^15^; (2) the experimental methods used for affinity evaluation (such as SPR, enzyme-linked immunosorbent assay (ELISA), and bio-layer interferometry (BLI)) are based on the hypothesis that the analyte samples contain homogeneous molecular species. However, IgG characteristics are intrinsically heterogeneous. Therefore, these methods may yield inconsistent results if the samples are highly heterogeneous. (3) IgG-FcRn interactions are pH-dependent. Therefore, an affinity measurement at a single pH is not sufficient as an indicator of PK properties. From a structural perspective, the detailed structure of the complex of full-length IgG and FcRn has not yet been revealed. Based on the crystal structure, several studies have reported that the Fab arms are located close to the membrane surface when IgG binds to FcRn, and the effect of Fab on IgG-FcRn binding has been studied^16–25^. Brinkhaus et al. discussed that Fab impairs FcRn binding due to this structural hindrance^26^. Moreover, several studies have shown that the amino acid sequence or surface charges in the variable region also affect IgG-FcRn affinity^11,12,27–31^. However, the molecular mechanisms by which domains other than Fc, or surface charges, affect the FcRn-binding remain unclear^25^.

Schlothauer et al. have reported an analytical FcRn-immobilized affinity chromatography^32^. This analytical technology is highly useful and widely used for in vitro FcRn affinity evaluation. In the current study, we developed an engineered FcRn-immobilized column for affinity chromatography. The thermal stability of the immobilized FcRn was increased to resist the harsh environment of liquid chromatography. Using the FcRn column, we analyzed methionine-oxidized antibody and FcRn affinity-engineered mutants. Moreover, we analyzed 13 therapeutic monoclonal antibodies to elucidate the mechanism of how the amino acid sequence in the variable region affects the FcRn affinity. Based on the results, we further investigated the mechanism of positive charges in the L chain affect FcRn affinity by mutation analysis. Through these studies, we elucidated the structural insights into IgG-FcRn interactions. In addition, although the correlation between the elution profile of the FcRn column and in vivo data was not addressed, it was demonstrated that the method is useful for quality assessment of therapeutic antibodies.

## Results

### Development of the FcRn column

**Fig. 1:**
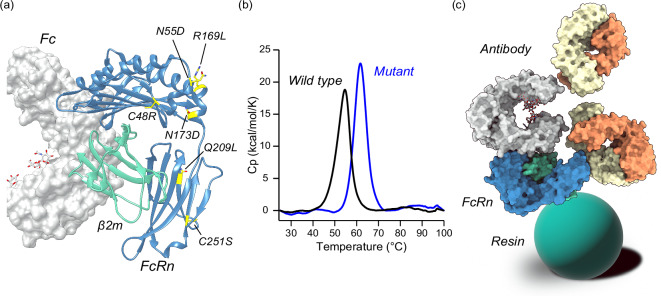
Design and development of the FcRn-immobilized column. **a** The mutation sites of FcRn are shown. The six mutation sites in FcRn are colored in yellow. K272E is not displayed in the figure because the structure of the FcRn C-terminus was not determined in the crystal structure. **b** Thermal stability of FcRn wild type and mutant. DSC thermogram of wild type (black) and mutant (blue) FcRn are shown. **c** A schematic view of IgG, FcRn, and resin is represented. The model was assembled using the crystal structures of human FcRn (steel blue), β_2_m (medium aquamarine), Fc (light gray), Fab H chain (light salmon), and Fab L chain (lemon chiffon). PDB IDs of each structure are as follows: adalimumab Fab, 4NYL; Fc, 4w4N; and FcRn, 4N0U. The image of the resin is shown as a sphere colored in teal.

The proteinaceous ligand for an affinity column must be sufficiently stable to resist the harsh environment (e.g., high salt concentration, high pH, low pH, and high column pressure). To obtain a suitable ligand for the affinity chromatography column, FcRn was mutated by error-prone polymerase chain reaction (PCR). The obtained mutant contained seven mutations: C48R, N55D, R169L, N173D, Q209L, C251S, and K272E. The mutation sites in FcRn are shown in Fig. 1a. The seven mutation sites in FcRn were distant from the Fc-binding site. Thus, the binding interaction between IgG and FcRn is not altered upon these mutations. The thermal stability of the wild type and the mutant FcRn was evaluated using differential scanning calorimetry (DSC) (Fig. 1b). The melting temperature (*Tm*) of the wild type was 54.1 °C, whereas that of the mutant was 61.7 °C. The purified mutant FcRn protein was immobilized on the surface of the resin (Fig. 1c).

### Analysis of methionine oxidized antibody using the FcRn column

**Fig. 2:**
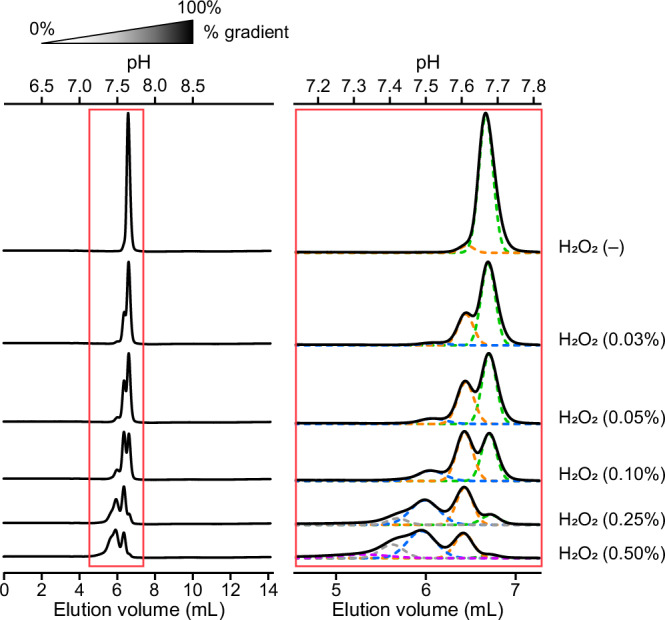
FcRn affinity chromatography of methionine oxidized antibody. An overall view of the FcRn affinity chromatography is shown on the left. A zoomed-in view of the FcRn affinity chromatography is shown on the right. The solid lines represent the chromatogram of absorbance at 280 nm. The concentration of H_2_O_2_ used for the oxidation is shown on the right side. The fitted curves for each peak are colored in green, orange, blue, gray, and purple. The % gradient and pH values are shown on top.

First, we evaluated the effect of methionine oxidation of antibodies on the FcRn interaction. It is well known that oxidation of Met252 and Met428 in the Fc region impairs FcRn-binding and decreases its long serum half-life. Stracke et al. showed that exposure to H_2_O_2_ resulted in the oxidation of Met252 and Met428^33^. We prepared oxidized adalimumab and analyzed using the developed FcRn column. The overall chromatograms of the oxidized adalimumab at each H_2_O_2_ concentration are shown in Fig. 2. The non-oxidized adalimumab showed a sharp peak at approximately pH 7.7. As the H_2_O_2_ concentration increased, several peaks appeared before the peak of non-oxidized adalimumab, and overall peaks became lower and broader.

### Analysis of FcRn affinity-engineered mutants

**Fig. 3:**
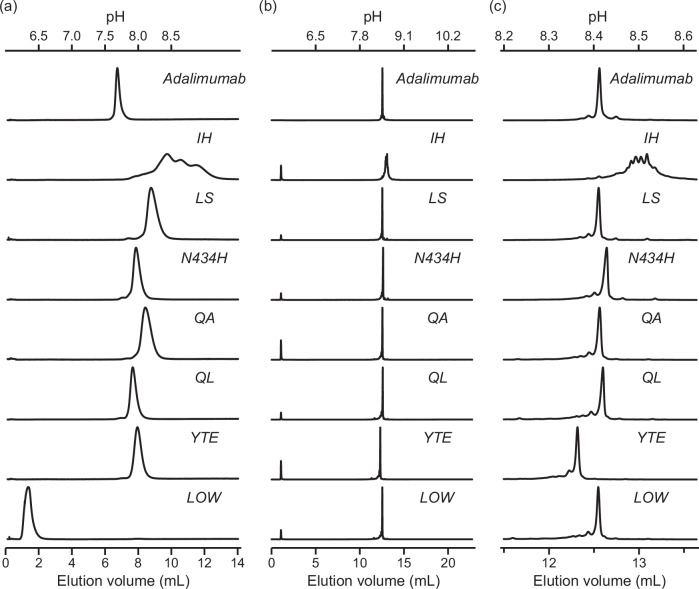
FcRn and ion-exchange chromatography analysis of FcRn affinity engineered mutants. **a** FcRn affinity chromatography of FcRn affinity engineered mutants. **b** Overall view of the ion-exchange chromatography. **c** A zoomed-in view of the ion-exchange chromatography. Solid lines represent the chromatogram of absorbance at 280 nm. The pH of elution is shown on top.

To assess the effects of FcRn affinity-engineering, we prepared seven mutants (six with increased affinity, and one with impaired affinity) and analyzed them using the FcRn column. The chromatograms are shown in Fig. 3a. The six mutants with increased affinity (IH, LS, N434H, QA, QL, and YTE) showed delayed peaks compared to adalimumab. In contrast, the null mutant (LOW) showed a markedly earlier elution (flow-through), indicating that the mutant was not bound to the FcRn column. The peaks of the eluted antibodies were almost single, except for the IH mutant. Interestingly, the IH elution peak was split into several broad peaks.

Ion-exchange chromatography was performed to assess the charge heterogeneity, especially for the IH mutant (Fig. 3b, c). The elution peaks reflected the overall charge of the antibody molecules. Thus, the order of antibody elution differed between the FcRn column and ion-exchange chromatography. Consistent with the results of FcRn affinity chromatography, only the IH mutant exhibited split and broad peaks.

### Analysis of pH-dependent FcRn affinity

**Fig. 4:**
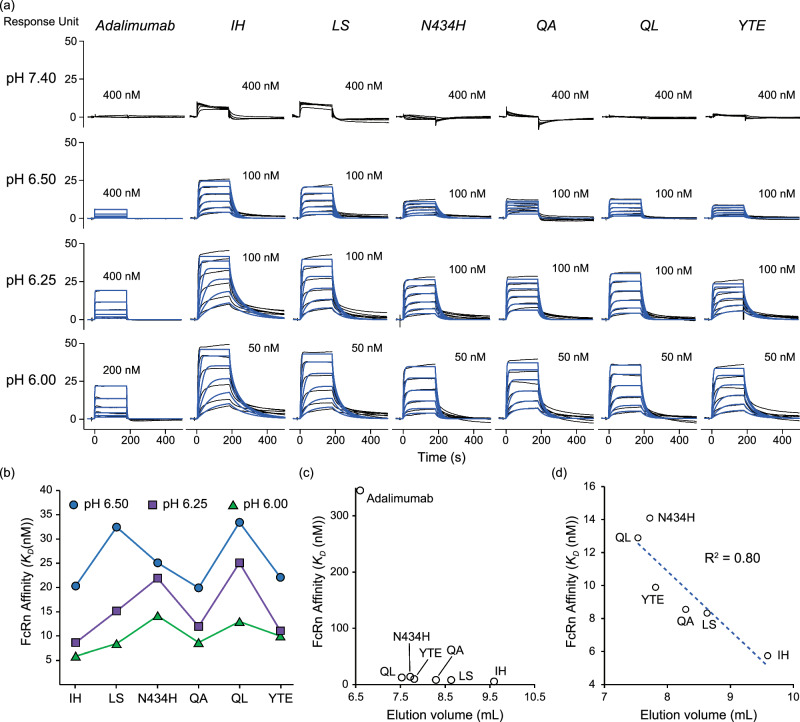
SPR analysis of adalimumab and its FcRn affinity-engineered mutants. **a** SPR sensorgrams of FcRn binding on the sensor chip. The measurements were performed at pH 7.40 (top), pH 6.50, pH 6.25, and pH 6.00 (bottom). The black solid lines represent sensorgrams. The blue solid lines represent fitted sensorgrams. The highest analyte (antibody) concentration is shown above each sensorgram. The sensorgrams at pH 7.4 could not be fitted using the kinetic model. **b** The determined affinity (dissociation constant, *K*_*D*_) is plotted. The affinity values at pH 6.50 (blue), pH 6.25 (magenta), and pH 6.00 (green) are shown. **c** The correlation between FcRn affinity at pH 6.0 versus elution volume of FcRn affinity chromatography is shown. **d** The correlation for FcRn affinity-engineered mutants and its R-squared value for linearity is shown.

FcRn affinity chromatography is a method to assess pH-dependent FcRn affinity of IgG. Since antibodies that elute excessively late in FcRn affinity chromatography are likely to bind FcRn strongly at neutral pH, and therefore cannot exhibit extended serum half-life, we considered it important to reveal the correlation between the pH dependence of the FcRn affinity and the elution profiles of FcRn affinity chromatography. As an orthogonal assessment, we measured the FcRn affinity of the FcRn affinity-engineered mutants using SPR at pH 6.00, 6.25, 6.50, and 7.40 (Fig. 4 and Supplemental Table 1). To decrease the influence of bivalent binding of FcRn on the sensor chip, the amount of immobilized FcRn was reduced^34^. Overall, the affinities of these mutants were decreased with increasing pH (Fig. 4b). At pH 7.40, while the IH and LS mutants showed weak binding to FcRn, the other antibodies (adalimumab, N434H, QA, QL, and YTE) did not bind to FcRn. In particular, for the mutants, the plot of affinity at pH 6.0 versus the elution volume showed a modest correlation (Fig. 4c, d).

### Analysis of 13 therapeutic monoclonal antibodies

**Fig. 5:**
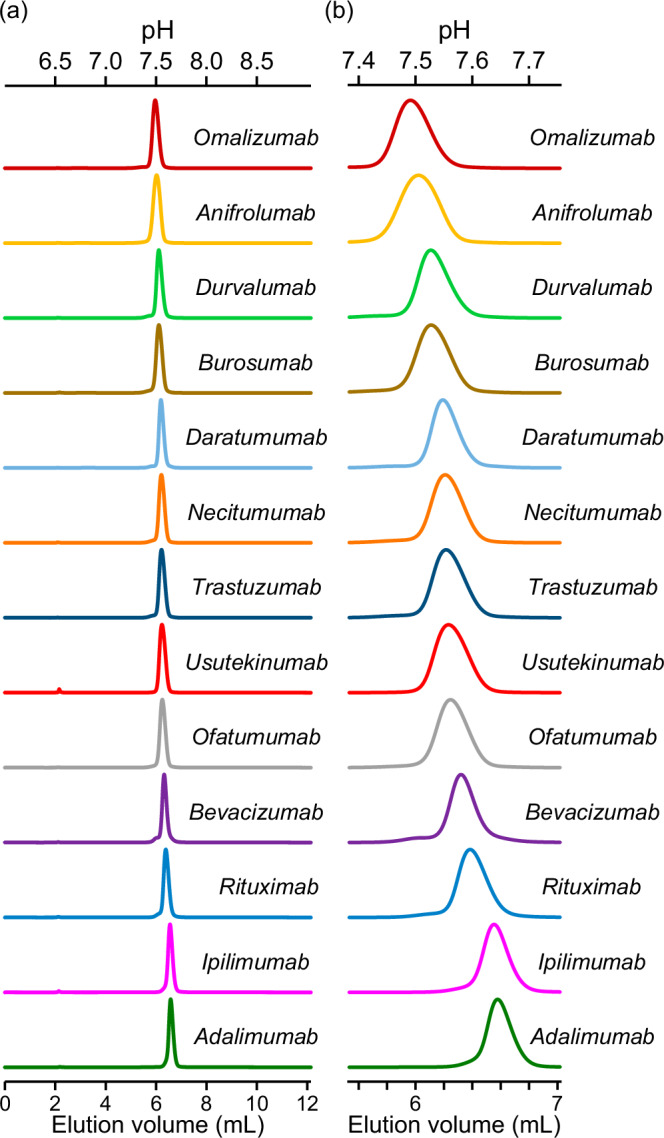
FcRn affinity chromatography of 13 therapeutic antibodies. **a** The FcRn affinity chromatography of therapeutic monoclonal antibodies is shown on the left. **b** A zoomed-in view of the chromatography. Solid lines represent the chromatogram of absorbance at 280 nm. The pH of elution is shown on top.

Multiple studies have shown that the charges of antibodies affect the FcRn affinity^12,27,29,31,35^. However, which part of IgG has an impact on FcRn affinity, and to what extent the impact differs among several antibodies required exploration. We analyzed 13 different therapeutic monoclonal antibodies using FcRn affinity chromatography (Fig. 5a, b). Although these 13 antibodies are all IgG1 (three mutations in anifrolumab (L234F, L235E, and P331S) are distant from the binding interface of Fc-FcRn), the elution profiles differed remarkably. Among these 13 antibodies, omalizumab eluted first, and adalimumab eluted last.

**Fig. 6:**
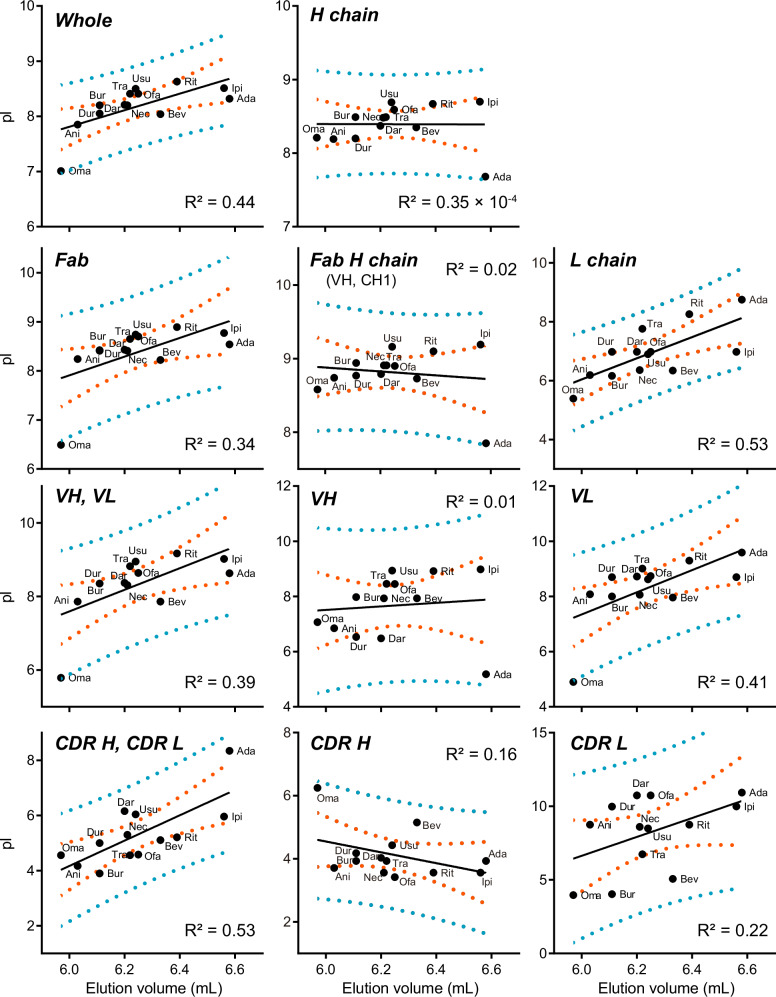
Correlation between pI and elution volume in FcRn affinity chromatography. The x-axis shows the elution volume in FcRn affinity chromatography. The y-axis represents the pI for each domain/chain. Orange dotted lines indicate the 95% confidence band. The turquoise dotted lines indicate the 95% prediction band. The plot was generated using GraphPad Prism software.

Furthermore, we assessed the correlation between the elution volume and the charges of each domain or chain of the antibodies. The amino acid sequence-based isoelectric point (pI) of each domain/chain was calculated and plotted (Fig. 6). The pI was calculated for the following domains/chains: whole, H chain, Fab, Fab H chain, L chain, VH and VL, VH, VL, CDR H and CDR L, CDR H, and CDR L. The highest R-squared value for linearity was 0.53, which was derived from the “L chain” and “CDR H and CDR L”. Overall, the R-squared values related to the L chain (L chain, 0.53; VL, 0.41; CDR L, 0.22) were higher than those for the H chain (H chain, 0.35 × 10^−4^; Fab H chain, 0.02; VH, 0.01; CDR H, 0.16). Although a clear correlation between the elution volume and pI was not observed, it is reasonable to conclude that the pI of the L chain, rather than that of the H chain, has an impact on FcRn affinity.

### Analysis of the charges in the L chain of antibodies

**Fig. 7:**
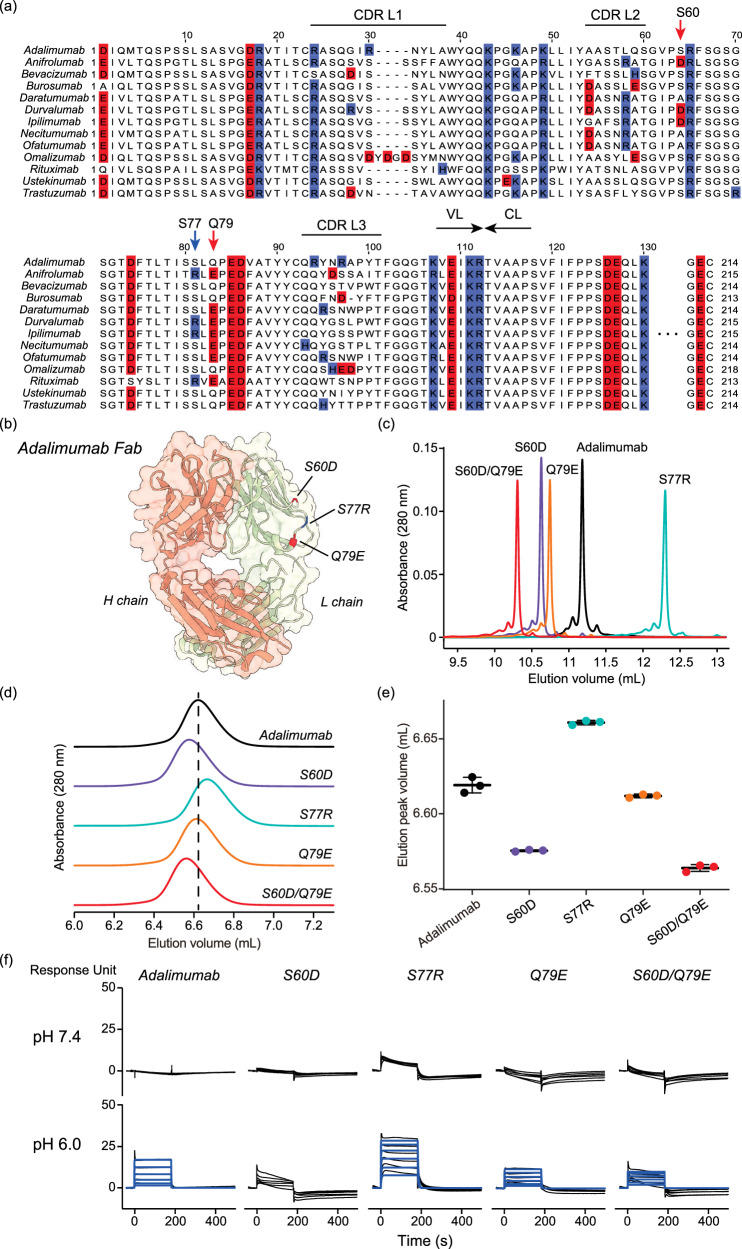
Analysis of the impact of charges in the L chain on FcRn affinity. **a** Comparison of amino acid sequences of the L chain for 13 antibodies. S60, S77, and Q79 (according to the numbering of adalimumab) are highlighted using arrows. The acidic amino acids are colored in red. The basic amino acids are colored in indigo blue. **b** The structure of adalimumab Fab is shown (PDB ID, 4NYL). **c** Ion exchange chromatography of adalimumab and charge-engineered mutants. **d** The FcRn affinity chromatography of adalimumab and charge-engineered mutants. **e** Elution volume of FcRn affinity chromatography of adalimumab and charge-engineered mutants. Error bars represent data from 3 independent experiments (*n* = 3). **f** SPR sensorgrams of FcRn binding on the sensor chip. The measurements were performed at pH 7.4 and pH 6.0. The black solid lines represent sensorgrams. The blue solid lines represent fitted sensorgrams. The sensorgrams at pH 7.4 could not be fitted using the kinetic model.

To focus on how the pI of the L chain affects FcRn affinity, we compared the amino acid sequences of the variable region of the L chain of the 13 antibodies (Fig. 7a). We observed that positively or negatively charged residues were located in variable regions other than the complementarity-determining region (CDR). In addition, the pattern of the charged residues varied depending on the antibody. Specifically, the Ser60 in adalimumab (numbered according to adalimumab) is an aspartic acid (negatively charged) in anifrolumab, durvalumab, and ipilimumab. The Ser77 in adalimumab is an arginine (positively charged) in anifrolumab, durvalumab, ipilimumab, and rituximab. The Gln79 in adalimumab is a glutamic acid (negatively charged) in anifrolumab, daratumumab, durvalumab, ipilimumab, necitumumab, ofatumumab, and rituximab. Thus, we hypothesized that the charged residues at these three positions might also affect FcRn affinity. These three residues are located on the lateral surface of the L chain (Fig. 7b).

To test this hypothesis, we prepared charge-engineered adalimumab mutants and performed FcRn affinity chromatography. S60, S77, and Q79 of adalimumab L chain were mutated to charged residues: S60D, S77R, and Q79E, respectively. Ion-exchange chromatography analysis showed that the negative charge-introduced mutants (S60D and Q79E) eluted earlier than the parent adalimumab (Fig. 7c). Additionally, the S60D/Q79E mutant eluted earlier than the S60D and Q79E. In contrast, the elution peak of the positive charge-introduced mutant (S77R) was delayed. These results confirmed that the charge of the adalimumab was modified by the mutagenesis. Subsequently, we performed the FcRn affinity chromatography using these adalimumab L chain mutants (Fig. 7d, e). Consistent with the results of ion-exchange chromatography, the elution of S60D was earlier than the parent adalimumab, that of Q79E was slightly earlier, that of S60D/Q79E was further earlier, and that of S77R was delayed. The SPR results also supported the increase or decrease in FcRn affinity upon these mutations (Fig. 7f, Supplemental Table 1). These results strongly indicated that the charge on the lateral surface of the L chain also affects FcRn affinity.

## Discussion

Multiple mechanisms, including phagocytosis, target-mediated internalization, and target-independent pinocytosis, are involved in the regulation of therapeutic antibody pharmacokinetics. In particular, the molecular interactions between therapeutic antibodies and FcRn affect the long serum half-life of antibodies. Thus, the in vitro FcRn affinity of IgG is an important indicator of its in vivo PK properties. However, the molecular basis of the IgG-FcRn interaction is not fully understood from experimental and structural perspectives. In this study, we developed an engineered FcRn-immobilized column. Using this FcRn column, we performed affinity chromatography and analyzed various types of antibodies to reveal the correlations between antibody characteristics and FcRn affinity. Furthermore, we focused on the effect of charges in the L chain on FcRn affinity from a structural perspective.

The FcRn affinity chromatography enabled an evaluation of FcRn affinity from the elution profile. The elution profiles of Met-oxidized antibodies and FcRn-affinity engineered antibodies were consistent with the previously reported results^11,32,33,36–38^. As the elution peaks of the Met-oxidized antibody were clearly separated, it is evident that this method possesses sufficient specificity as a performance characteristic to identify Met-oxidation of antibodies. The elution profiles of antibodies reflected their affinity for FcRn. Meanwhile, the antibody with abrogated binding to FcRn flows through the column without being captured. Hence, in contrast to SPR, FcRn affinity chromatography can be used to determine the molecular populations in samples based on FcRn affinity. Borrok et al. reported that the high affinity for FcRn at both acidic and neutral pH leads to rapid serum clearance^10^. To achieve FcRn-mediated half-life extension, antibody molecules must be released from FcRn when the antibodies are recycled back into the blood circulation. Given this, FcRn affinity evaluation at a single pH (e.g., pH 6.0) is not sufficient to indicate PK properties. Since the most important parameter to be evaluated is pH-dependent FcRn affinity, the pH gradient-based FcRn affinity chromatography is a highly effective methodology.

Using the FcRn column, we showed that the elution profiles varied depending on the antibodies, indicating that the sequence of the Fab region affected FcRn affinity. Given that the Fab arms are accommodated on the membrane surface when IgG binds to FcRn, as reported by Brinkhaus et al., it is reasonable that Fab also interacts with FcRn^26^. Furthermore, we showed that the charges in the L chain, rather than those in the H chain, affected FcRn affinity. These results were consistent with previous studies^11,12,27–31^. Since the involvement of the Fab domain in the IgG-FcRn interaction is not a highly specific interaction, the relationship between the coordinates of positive charges (i.e., their quantity and positions) and the FcRn affinity remains unclear. However, our study showed that the effect of positive charges on FcRn affinity is not solely dependent on the positive charges of a particular domain, such as Fab, VL, or CDR. The mutation analysis suggested that the charges on the lateral surface of the L chain also affect FcRn affinity.

**Fig. 8:**
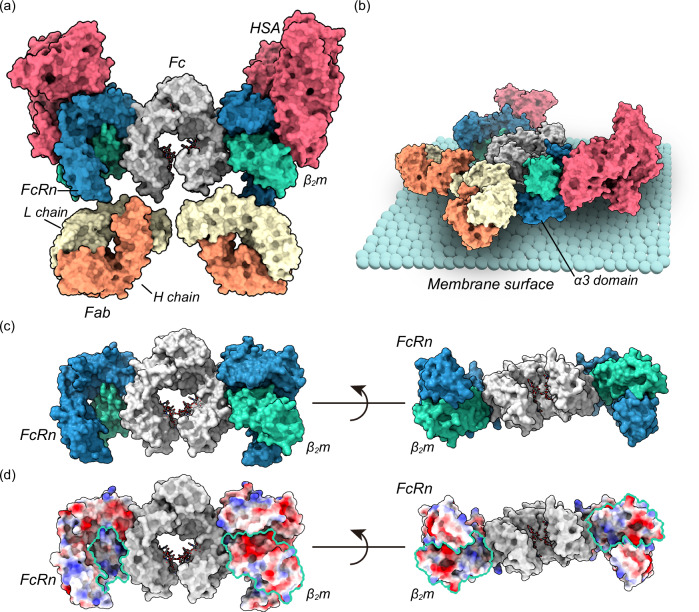
Structural model of full-length IgG and FcRn complex. **a** The assembled crystal structures of the complex of adalimumab Fab, Fc, FcRn, β_2_m, and HSA. PDB IDs of each structure are as follows: adalimumab Fab, 4NYL; Fc, 4w4N; and FcRn-β_2_m, 4N0U; and HSA, 4N0U. Each molecule is colored as follows: Fab H chain, light salmon; Fab L chain, lemon chiffon; Fc, light gray; FcRn, steel blue; β_2_m, medium aquamarine; and HSA, pale violet red. **b** Top and side view of the model of the complex on membrane surface. **c** Top view (left) and rotated view (right) of the Fc-FcRn complex. **d** Positively charged, negatively charged, and neutral residues in the FcRn and β_2_m are colored blue, red, and white, respectively. The models were generated by aligning the crystal structures using UCSF Chimera and ChimeraX.

Based on these findings, we assembled a structural model of the complex of full-length IgG, FcRn, and human serum albumin (HSA) using the crystal structures (Fig. 8). Multiple studies have discussed the IgG-FcRn binding model on the membrane surface, including “standing-up”, “lying down”, “Y-shape”, “T-shape”, and “reclined model”^22–24,26^. We speculated the “reclined model” is reasonable considering that (1) the binding stoichiometry between IgG and FcRn is possibly 1:2, (2) the structural clash between the Fab arm and membrane surface should be avoided, and (3) FcRn plays an important role in salvaging and maintaining HSA in blood circulation, thus the simultaneous binding of HSA and IgG to FcRn should not be compromised^22,39^. In our model, the lateral surface of the L chain touches the bottom of the α3 domain of FcRn (or C-terminus near the membrane surface) and β_2_m. The model indicated that multiple negatively charged residues (shown in red) are present at the bottom region of FcRn and β_2_m. These negatively charged residues may interact with the Fab of IgG. Thus, this model may explain the mechanism by which charges in the L chain affect the FcRn affinity. In contrast, an elevated pI of IgG (positively charged) results in a shortened serum half-life^7,35,40,41^. This is presumably because the positively charged IgG can be captured by the negatively charged cell surface, resulting in increased nonspecific cellular uptake (clearance). Considering the contradiction between FcRn affinity-derived serum half-life extension and the non-specific cellular uptake-derived rapid clearance, the coordinates of positive charges (quantity and positions) are remarkably important.

Estimating the PK properties of therapeutic antibodies in humans remains challenging because multiple parameters, such as FcRn affinity of the antibody, administration dose, administration route, patient body weight, properties of target molecules, and underlying conditions, affect PK properties. Therefore, extensive efforts have been made to understand the correlation between FcRn affinity and PK properties. Schlothauer et al., Grevys et al., and Gjølberg et al. conducted comprehensive analyses, including affinity measurements, FcRn affinity column chromatography, in vitro cell-based assays, and experiments using human FcRn transgenic mice (in vivo)^11,21,32,37^. The results obtained using human FcRn transgenic mice did not always correlate with those of other experiments. As discussed in the literature, in vivo studies using human FcRn transgenic mice pose a few issues, including non-specific cellular uptake and ADA^11,13^. Grevys et al. revealed that a highly positively charged anti-IL 12/23 p40 antibody (briakinumab) had a shorter serum half-life than that of anti-IL 12/23 p40 antibody (ustekinumab), even though the elution of briakinumab by FcRn affinity chromatography was markedly later than that of ustekinumab^11^. As discussed above, this was presumably due to the rapid clearance of positively charged IgG via nonspecific cellular uptake. Furthermore, FcRn-mediated antibody recycling is a dynamic and complex event at the membrane surface that involves a heterogeneous IgG:FcRn binding ratio (1:1 to 1:2) and a transition from planar to tubular membrane structures^42,43^. Therefore, our FcRn affinity chromatography-based method is useful mainly for FcRn affinity analysis (quality assessment), and it remains challenging to extrapolate the results of FcRn affinity chromatography to in vivo/in human PK properties in some cases. Nevertheless, the progressive accumulation of knowledge regarding the structure and affinity of IgG-FcRn interactions could pave the way for more accurate prediction of PK properties.

Meanwhile, we were unable to determine the structure of the IgG-FcRn complex using transmission electron microscopy (TEM), even at low-to-medium resolution. Sun et al. visualized the IgG-FcRn complex using electron microscopy and discussed the Fab-FcRn interactions based on the conformation of IgG^44^. An increase in the resolution of the structure of the IgG-FcRn complex will unquestionably advance our understanding of IgG-FcRn interactions.

In conclusion, we elucidated structural insights into IgG-FcRn interactions using engineered FcRn-immobilized affinity chromatography. We hope that this study will lead to better and effective characterization methods for therapeutic antibodies. Moreover, we hope that our findings will encourage the development of novel molecular designs of therapeutic antibodies.

## Materials and methods

### Design of the FcRn column

Thermally stabilized human FcRn α-chain mutants were obtained as described previously^45^. Briefly, using the human FcRn α-chain gene as a template, error-prone PCR was performed to introduce one or two amino acid substitutions, thereby generating an FcRn mutant library. Subsequently, mutant FcRn proteins were expressed in *Escherichia coli* strain BL21 DE3 (NIPPON GENE, Cat# 312-06534) using the FcRn mutant library. FcRn mutants with improved thermal stability were identified based on their residual activity after heat treatment. By combining multiple mutations, an engineered human FcRn mutant with enhanced thermal stability was obtained.

The protein immobilized onto the resin included a human FcRn α-chain and β_2_m. The sequence of human FcRn α-chain was linked at the C-terminus of human β_2_m via a 5 × (GGGGS) linker. In other words, the protein consisting of β_2_m-GGGGS linker-FcRn (hereinafter referred to as FcRn) was immobilized on a TSKgel resin (particle size, 10 μm). The FcRn protein was expressed using *E. coli* strain BL21 (DE3) transformed with an expression vector. The cells were harvested and lysed using buffer (50 mM Bis-Tris Propane, 2.4 mM MgSO_4_, 150 mM NaCl, 5 units/mL benzonase, 0.006% (w/v) lysozyme, 0.6% (v/v) Triton X-100, pH 10.0). The extracted proteins were purified using Ni-NTA agarose (Wako, Cat# 143-09763) and IgG-Sepharose (Cytiva, Cat# 17061801). The C-terminus of FcRn was immobilized using an optimized, highly oriented coupling method. The amino acid sequence is shown in Supplemental Fig. 1. The FcRn-immobilized resins were packed in a solid support (4.6 mm I.D. × 50 mm). The analytical performance of the column was retained beyond 200 uses.

### Differential scanning calorimetry (DSC)

The thermal stability of FcRn was determined using a PEAQ-DSC instrument (Malvern, UK). Wild-type FcRn was purchased from Gamma Proteins, Ltd. (Cat# HUFCRN-U). Mutant FcRn was the protein expressed and purified in-house. Protein samples were concentrated and buffer-exchanged with 50 mM phosphate, 150 mM NaCl (pH 6.0). The protein samples at a concentration of 10 μM were subjected to DSC measurements from 25 to 100 °C at a scan rate of 90 °C/h. The thermograms were normalized by subtracting the signal from the reference cell containing only buffer. The melting temperatures (*Tm*) were calculated by a standard fitting procedure using an evaluation software using a non-two-state model.

### FcRn affinity chromatography

The FcRn affinity chromatography was performed using an ÄKTA Avant 25 (Cytiva, Sweden). Mobile phase A was 50 mM 2-morpholinoethanesulfonic acid (MES), 150 mM NaCl (pH 6.5), and mobile phase B was 50 mM Tris-HCl (pH 8.5). A total of 50 µg of antibody sample was diluted at least 10-fold with the mobile phase A to adjust the pH. A linear gradient of buffer B (0–100%) at a flow rate of 0.4 mL/min was applied to the FcRn column for 20 min to elute the antibody. The elution positions of the peaks in the chromatograms were determined based on the elution volume of the largest peak. The fitting of the chromatogram was performed using the Microsoft Excel Solver tool to minimize the differences between the data and the model.

### Methionine oxidation

Adalimumab samples were subjected to H_2_O_2_ according to the protocols to induce methionine oxidation in the Fc region^33,46–48^. Adalimumab (Humira®) was purchased from Japanese pharmaceutical distributors. Adalimumab samples were buffer-exchanged and diluted to 1 mg/mL in a buffer containing 50 mM MES, 150 mM NaCl (pH 6.5). H_2_O_2_ was added to the antibody samples at 0.03, 0.05, 0.1, 0.25, and 0.5%. The reaction mixture was incubated for 17 h at 4 °C. The oxidation reactions were stopped by adding a buffer containing 5 mM L-methionine. L-methionine in the quenched sample was subsequently removed by buffer exchange using Amicon Ultra (Merck, Cat# UFC901024).

### Preparations of adalimumab mutants

We prepared seven adalimumab mutants whose FcRn affinities were engineered in previous studies. The mutants were as follows: IH (P257I/N434H), LS (M428L/N434S), N434H, QA (T307Q/N434A), QL (T250Q/M428L), YTE (M252Y/S254T/T256E), and LOW (I253A/ H310A/H435A)^19,49–55^. The adalimumab wild type used in this study was derived from a therapeutic drug (Humira®), not prepared in-house. Expression vectors encoding the adalimumab variants were constructed in the previous study^38^. Antibodies were produced by using the Expi293 expression system (ThermoFisher Scientific, Cat# A14635). Expi293 cells (50 mL culture) were transfected using 50 µg pFuse vector (25 µg of H chain and 25 µg of L chain) and ExpiFectamin 293 Transfection Kit (ThermoFisher Scientific, Cat# A14525). After 6 days of cultivation, the supernatant was collected by centrifugation (40,000 × *g*, 30 min). The obtained supernatant was filtered through a 0.22 μm bottle top filter (Corning, Cat# 430626) and was subjected to protein A purification. The mutant LOW was subjected to protein L purification. After washing the column with 20 mM Tris-HCl, 150 mM NaCl (pH 7.2), the antibody samples were eluted with 50 mM glycine (pH 3.0) and neutralized with 1 M Tris-HCl (pH 8.0). The eluted antibody samples were further purified by size exclusion chromatography using a HiLoad 26/60 Superdex 200-pg column (Cytiva) equilibrated with 20 mM histidine, 80 mM NaCl (pH 6.0). Expression vectors of the charge-engineered mutants were prepared by Sino Biologicals (Beijing, China). The charge-engineered mutants were expressed as described above for the FcRn affinity-engineered mutants.

### Ion exchange chromatography

Ion exchange chromatography was performed using a BioPro IEX SF 4.6 mm I.D. × 100 mm column (YMC, Cat# SF00S03-1046WP). Mobile phase A was CX-1 pH Gradient Buffer A (pH 5.6) (ThermoFisher Scientific, Cat# 085346), and mobile phase B was CX-1 pH Gradient Buffer B (pH 10.2) (ThermoFisher Scientific, Cat# 085348). A total of 50 µg of antibody sample was diluted at least 10-fold with mobile phase A to adjust the pH, and then the sample was applied to a Waters ACQUITY UPLC H-class system. A linear gradient of mobile phase B (0–100%) at a flow rate of 0.3 mL/min was applied to the column for 60 min to elute the antibody.

### Surface plasmon resonance (SPR)

A Biacore 8 K instrument (Cytiva) was used to evaluate the binding of antibodies to FcRn. An Avi-tagged recombinant human FcRn-β_2_m was purchased (ACROBiosystems, Cat# FCM-H82W4). FcRn was immobilized on the CAP chip using the Biotin CAPture Kit (Cytiva, Cat# 28920234). FcRn (0.3 µg/mL) was injected for 60 sec, and the aimed immobilized level was 20 RU. The running buffer was 50 mM phosphate, 150 mM NaCl (pH 6.0). The regeneration solution was 20 mM Tris-HCl, 150 mM NaCl (pH 7.4). The association and dissociation time was 180 and 300 s, respectively. The concentrations of antibodies were as follows. The adalimumab was diluted using the running buffer by 2-fold serial dilutions from 400 to 12.5 nM. The adalimumab mutants were diluted using the running buffer in 2-fold serial dilutions from 100 to 3.1 nM.

### Therapeutic monoclonal antibody sample

Adalimumab (Humira®), anifrolumab (Saphnelo®), bevacizumab (Avastin®), burosumab (Crysvita®), daratumumab (Darzalex®), durvalumab (Imfinzi®), ipilimumab (Yervoy®), necitumumab (Portrazza®), ofatumumab (Kesimpta®), omalizumab (Xolair®), rituximab (Rituxan®), trastuzumab (Herceptin®), and ustekinumab (Stelara®) were purchased from Japanese pharmaceutical distributors.

### Amino acid sequence-based isoelectric point (pI) calculation

The amino acid sequence-based pI of the antibodies was calculated using the ProtParam tool (ExPASy)^56^.

### Statistics and reproducibility

Error bars represent standard deviation. At least three experiments were conducted for SPR and chromatography using independently prepared samples.

### Reporting summary

Further information on research design is available in the Nature Portfolio Reporting Summary linked to this article.

## Supplementary information

Supplemental Information

Description of Additional Supplementary Files

Supplemental data

Reporting Summary

## Data Availability

The sequence of FcRn was obtained from UniProt entry; P55899 FCGRN_HUMAN. All data are available in the article itself and its Supplemental data. All other data are available from the corresponding author on reasonable request.
