# Supplementary material for: Integrative structural insights into the IgG-FcRn interactions revealed by engineered FcRn-immobilized affinity chromatography
Source: Commun Biol. 2026 Mar 3;9:513. doi: 10.1038/s42003-026-09789-3 (PMC13066580; doi:10.1038/s42003-026-09789-3)
Supplement: Supplementary file 1 — Supplemental Information [file 42003_2026_9789_MOESM1_ESM.pdf]

Supplemental Information; Integrative structural insights into IgG-FcRn interactions revealed by engineered FcRn-immobilized affinity chromatography

Masato Kiyoshi[1,*], Takuo Suzuki[1], Naruaki Inoue[2], Ryoko Otake[2], Tatsuya Yumoto[2], Linko Hirono[2], Yosuke Terao[2], Hiroko Shibata[1], Teruhiko Ide[2], Satoru Nagatoishi[3,4], Kouhei Tsumoto[3,4], and Akiko Ishii-Watabe[1]

[1]Division of Biological Chemistry and Biologicals, National Institute of Health Sciences, Kawasaki, Kanagawa, Japan.[2]Life Science Research Laboratory, Tosoh Corporation, Ayase, Kanagawa, Japan. [3]The Institute of Medical Science, The University of Tokyo, Minato-ku, Tokyo, Japan. [4]The Department of Bioengineering, School of Engineering, The University of Tokyo, Bunkyo-ku, Tokyo, Japan.

*Correspondence and requests for materials should be addressed to K.M. (email: m.kiyoshi@nihs.go.jp)

```
         10        20        30        40        50
IQRTPKIQVYSRHPAENGKSNFLNCYVSGFHPSDIEVDLLKNGERIEKVE
         60        70        80        90       100
HSDLSFSKDWSFYLLYYTEFTPTEKDEYACRVNHVTLSQPKIVKWDRDMG
        110       120       130       140       150
GGGSGGGGSGGGGSGGGGSGGGGSAESHLSLLYHLTAVSSPAPGTPAFWV
        160       170       180       190       200
SGWLGPQQYLSYNSLRGEAEPRGAWVWEDQVSWYWEKETTDLRIKEKLFL
        210       220       230       240       250
EAFKALGGKGPYTLQGLLGCELGPDNTSVPTAKFALNGEEFMNFDLKQGT
        260       270       280       290       300
WGGDWPEALAISQRWQQQDKAANKELTFLLFSCPHRLREHLELGRGDLEW
        310       320       330       340       300
KEPPSMRLKARPSSPGFSVLTCSAFSFYPPELLLRFLRNGLAAGTGQGDF
        360       370       380       390
GPNSDGSFHASSSLTVKSGDEHHYSCIVQHAGLAQPLRVELESPAESS
```

Supplemental Figure 1. Amino acid sequence of FcRn-β2m.
The amino acid sequence is shown (β_2m, blue; GGGGS linker, black; FcRn, green; and the mutated residues, red).

Supplemental Table 1. The affinity of adalimumab and affinity-engineered mutants for FcRn determined using SPR.

	**Affinity (K_D, nM)**			
pH	6.00	6.25	6.50	7.40
Adalimumab	345.0	813.0	36700.0	N.D.
IH	5.8	8.5	20.3	N.D.
LS	8.3	15.1	32.5	N.D.
N434H	14.1	21.9	25.1	N.D.
QA	8.6	11.9	19.9	N.D.
QL	12.9	25.1	33.5	N.D.
YTE	9.9	11.0	22.1	N.D.
LOW	N.D.	N.D.	N.D.	N.D.

Supplemental Table 2. The affinity of adalimumab and the charge-engineered mutants for FcRn determined using SPR.

	**Affinity (K_D, nM)**	
pH	6.0	7.4
Adalimumab	219	N.D.
S60D	N.D.	N.D.
S77R	38	N.D.
Q79E	343	N.D.
S60D/Q79E	942	N.D.
