# Supplementary material for: Integrative structural insights into the IgG-FcRn interactions revealed by engineered FcRn-immobilized affinity chromatography
Source: Commun Biol. 2026 Mar 3;9:513. doi: 10.1038/s42003-026-09789-3 (PMC13066580; doi:10.1038/s42003-026-09789-3)
Supplement: Supplementary file 2 — Description of Additional Supplementary Files [file 42003_2026_9789_MOESM2_ESM.pdf]

Description of Additional Supplementary files

File name: Supplementary Data

Description: Source data for figures used in the study.
